# Idebenone has preventative and therapeutic effects on pulmonary fibrosis via preferential suppression of fibroblast activity

**DOI:** 10.1038/s41420-019-0226-y

**Published:** 2019-11-18

**Authors:** Toshifumi Sugizaki, Ken-ichiro Tanaka, Teita Asano, Daisuke Kobayashi, Yuuki Hino, Ayaka Takafuji, Mikako Shimoda, Kaoru Mogushi, Masahiro Kawahara, Tohru Mizushima

**Affiliations:** 1 Department of System Chemotherapy and Molecular Sciences, Division of Bioinformatics and Chemical Genomics, Graduate School of Pharmaceutical Sciences, Kyoto University, Kyoto, Japan; 2 Laboratory of Bio-Analytical Chemistry, Research Institute of Pharmaceutical Sciences, Faculty of Pharmacy, Musashino University, 1-1-20 Shinmachi, Nishi-Tokyo, 202-8585 Japan; 3 Institute of Medical Science, St. Marianna University School of Medicine, 2-16-1, Sugao, Miyamae-ku, Kawasaki 216-8512 Japan; 4 Intractable Disease Research Center, Juntendo University Graduate School of Medicine, Tokyo, Japan; 5 LTT Bio-Pharma Co., Ltd, Shiodome Building 3F, 1-2-20 Kaigan, Minato-ku, Tokyo 105-0022 Japan

**Keywords:** Respiratory tract diseases, Drug development

## Abstract

Alveolar epithelial injury induced by reactive oxygen species (ROS) and abnormal collagen production by activated fibroblasts (myofibroblasts) is involved in the onset and exacerbation of idiopathic pulmonary fibrosis (IPF). Compared with alveolar epithelial cells, lung fibroblasts, especially myofibroblasts, exhibit an apoptosis-resistance phenotype (apoptosis paradox) that appears to be involved in IPF pathogenesis. Thus, we screened for chemicals eliciting preferential cytotoxicity of LL29 cells (lung fibroblasts from an IPF patient) compared with A549 cells (human lung alveolar epithelial cell line) from medicines already in clinical use. We identified idebenone, a synthetic analogue of coenzyme Q10 (CoQ_10_, an antioxidant) that has been used clinically as a brain metabolic stimulant. Idebenone induced cell growth inhibition and cell death in LL29 cells at a lower concentration than in A549 cells, a feature that was not observed for other antioxidant molecules (such as CoQ_10_) and two IPF drugs (pirfenidone and nintedanib). Administration of idebenone prevented bleomycin-induced pulmonary fibrosis and increased pulmonary ROS levels. Importantly, idebenone also improved pulmonary fibrosis and lung function when administered after the development of fibrosis, whereas administration of CoQ_10_ similarly prevented bleomycin-induced pulmonary fibrosis, but had no effect after its development. Administration of idebenone, but not CoQ_10_, suppressed bleomycin-induced increases in lung myofibroblasts. In vitro, treatment of LL29 cells with idebenone, but not CoQ_10_, suppressed TGF-β–induced collagen production. These results suggest that in addition to antioxidant activity, idebenone exerts inhibitory activity on the function of lung fibroblasts, with the former activity being preventative and the latter therapeutic for bleomycin-induced fibrosis. Thus, we propose that idebenone may be more therapeutically beneficial for IPF patients than current treatments.

## Introduction

Idiopathic pulmonary fibrosis (IPF) is a progressive and devastating chronic lung disease with an ultimately poor prognosis: the average survival time from diagnosis is 2.8–4.2 years^1,2^. Although steroids and immunosuppressants have long been used for treatment of IPF, it has been determined that these treatments cannot improve the prognosis of IPF patients^2–4^. Recently, two drugs, pirfenidone and nintedanib, have been shown to significantly improve the reduction of forced vital capacity (FVC) in clinical trials of IPF patients^2,5,6^. However, both of these drugs have serious adverse effects, especially within the gastrointestinal tract^5,6^. Thus, safer drugs eliciting a therapeutic effect equal to or higher than that of these two approved drugs are necessary.

IPF appears to be triggered by lung epithelial injury resulting from increased production of reactive oxygen species (ROS) and the subsequent repair and remodelling process, such as collagen synthesis, induced to address this injury. However, in IPF patients this process is excessively stimulated, resulting in abnormal fibrosis (abnormal wound repair and remodelling) characterised by collagen deposition^7,8^. As ROS can stimulate collagen synthesis, it is believed that ROS play a pivotal role in IPF pathogenesis. Supporting this notion, a number of antioxidant molecules, such as N-acetylcysteine (NAC) and superoxide dismutase (SOD), have shown beneficial effects in IPF patients and animal models of IPF, such as bleomycin-induced pulmonary fibrosis^9,10^.

Myofibroblasts produce and secrete excessive extracellular matrix (ECM) proteins, and are involved in abnormal wound repair and remodelling^11^. Myofibroblasts are primarily produced by transdifferentiation of peribronchiolar and perivascular fibroblasts in response to various stimuli, especially transforming growth factor (TGF)-β1^12^. Thus, compounds that suppress myofibroblast production or inhibit myofibroblast activity are good candidates for drugs to treat IPF patients. In fact, both pirfenidone and nintedanib reportedly suppress the transdifferentiation of fibroblasts into myofibroblasts and collagen production in activated fibroblasts^13–15^.

One possible mechanism for abnormal fibrosis in IPF patients is the “apoptosis paradox”. In IPF patient lungs, apoptosis is preferentially observed in alveolar epithelial cells rather than fibroblasts, and this unbalance of apoptosis is believed to be involved in IPF pathogenesis^16,17^. This characteristic is reproduced in vitro, as compared with lung epithelial cells, lung fibroblasts (especially those from IPF patients) showed resistance to apoptosis by various stressors^16,18^. Thus, chemicals that improve this balance are also good candidates for drugs to treat IPF patients.

As unexpected adverse effects of potential drugs are often revealed at later clinical trial stages, the number of newly developed drugs is decreasing. Thus, we are implementing a breakthrough strategy for drug discovery and development, focusing on the use of drugs in current clinical use for new indications^19^. A major advantage of this strategy is that the safety of these drugs is already well understood, which substantially reduces the risk of unexpected side effects in humans^19^. In previous studies, we screened drugs against IPF, chronic obstructive pulmonary disease (COPD) and functional dyspepsia according to this strategy and found effective drugs for each disease^20–22^. Therefore, we believe that this strategy is useful for discovering new candidates for treating human diseases.

In this study, we screened for compounds capable of inhibiting the growth of lung fibroblasts (LL29 cells) more potently than lung alveolar epithelial cells (A549 cells), and identified idebenone from a library of medicines already in clinical use. Intratracheal administration of idebenone, which has previously been used clinically as a brain metabolic stimulant, suppressed bleomycin-induced pulmonary fibrosis, alteration of lung mechanics and increases in pulmonary ROS levels. These results suggest that in addition to antioxidant activity, idebenone has inhibitory activity on the function of lung fibroblasts, with both of these activities contributing to its inhibitory effect on pulmonary fibrosis. Thus, we propose that idebenone may be therapeutically beneficial for IPF patients and have fewer side effects.

## Materials and methods

### Chemicals and animals

Idebenone, coenzyme Q10 (CoQ_10_), edaravone, α-tocopherol and pirfenidone were from Tokyo Chemical Industry (Tokyo, Japan), and nintedanib was from Cayman Chemical (Ann Arbor, MI). Bleomycin was from Nippon Kayaku (Tokyo, Japan), Sircol™ Collagen assay kit was from Biocolor (Antrim, UK), and chloral hydrate was from Nacalai Tesque (Kyoto, Japan). NAC, Chloramine T, 3-(4,5-dimethylthiazol-2-yl)-2,5-diphenyltetrazolium bromide (MTT), 4-(dimethylamino)-benzaldehyde (DMBA), potassium dichromate, phosphotungstic acid, phosphomolybdic acid, Orange G and acid fuchsin were obtained from Sigma (St. Louis, MO). Fetal bovine serum (FBS) was purchased from BioWest (Nuaille, France), and Ham’s F-12K (Kaighn’s modification) medium and SuperSignal West Dura Extended Duration Substrate was from Thermo Fischer Scientific (Waltham, MA). An antibody against α-smooth muscle actin (α-SMA) was from Abcam (Cambridge, Cambridgeshire), and an antibody against 8-hydroxy-2′-deoxyguanosine (8-OHdG) was from Japan Institute for the Control of Aging (Shizuoka, Japan). Recombinant human TGF-β1 were from R&D Systems (Minneapolis, MN), the assay kit for lactate dehydrogenase (LDH) was from Promega (Madison, WI), while thiobarbituric acid reactive substance (TBARS) Microplate Assay Kit was from Oxford Biomedical Research (Oxford, MI). Trypan blue, and Alexa Fluor 594 goat anti-rabbit immunoglobulin G were from Invitrogen (Carlsbad, CA). Isoflurane, Dulbecco’s modified Eagle’s medium (DMEM), l-hydroxyproline, sodium acetate, trichloroacetic acid (TCA), perchloric acid, azophloxin, aniline blue and formalin neutral buffer solution were obtained from WAKO Pure Chemicals (Tokyo, Japan). Mounting medium for immunohistochemical analysis (VECTASHIELD) was purchased from Vector Laboratories (Burlingame, CA), while Mayer’s haematoxylin, 1% eosin alcohol solution, mounting medium for histological examination (malinol) and Weigert’s iron haematoxylin were from MUTO Pure Chemicals (Tokyo, Japan). 4,6-diamidino-2-phenylindole (DAPI) was purchased from Dojindo (Kumamoto, Japan). Xylidine ponceau was from WALDECK GmbH & Co. KG, DIVISION CHROMA (Muenster, Germany). The Envision kit was from DAKO Co. (Carpinteria, CA), and Streptavidin-Biotinylated Horseradish Peroxidase Complex was from GE Healthcare (Buckinghamshire, UK). The RNeasy kit was obtained from Qiagen (Valencia, CA), the PrimeScript® 1st strand cDNA Synthesis Kit was from TAKARA Bio (Ohtsu, Japan), and the SsoAdvanced Universal SYBR Green Supermix was from Bio-Rad (Hercules, CA). ICR mice (6–7 weeks old, male) were purchased from Charles River (Yokohama, Japan). The experiments and procedures described here were carried out in accordance with the Guide for the Care and Use of Laboratory Animals as adopted and promulgated by the National Institutes of Health, and were approved by the Animal Care Committee of Musashino University.

### Treatment of mice with bleomycin and idebenone

Mice were anaesthetised with isoflurane and intratracheally administered either bleomycin (1 mg/kg or 2 mg/kg, once only) or idebenone (various doses) in sterile saline via a single channel pipette (P200). The first administration of idebenone was performed 1 h before bleomycin administration (except for experiments to examine the therapeutic effect).

### Real-time RT-PCR analysis

Total RNA was extracted from lung tissues using an RNeasy kit (Qiagen, Hilden, Germany) according to the manufacturer’s protocol. Using a PrimeScript First Strand cDNA Synthesis Kit (Takara Bio, Ohtsu, Japan), samples were reverse-transcribed and SsoAdvanced Universal SYBR Green Supermix, Bio-Rad’s CFX96™ Real-time system, and CFX Manager™ software (Hercules, CA) were used for real-time RT-PCR experiments. Electrophoretic analysis of reaction products was done to confirm specificity. Glyceraldehyde-3-phosphate dehydrogenase (GAPDH) cDNA was used as an internal standard. Primers were designed using either Primer3 or Primer-BLAST. Primer sequences will be provided upon request.

### Cell culture

A549 cells (human lung epithelial cell line) or LL29 cells (lung fibroblasts from IPF patient) were cultured in DMEM supplemented with 10% FBS or Ham’s F-12K (Kaighn’s Modification) medium supplemented with 15% FBS, respectively, in a humidified atmosphere of 95% air with 5% CO_2_ at 37 °C.

MTT assay^23^ or trypan blue staining assay with Countess® Automated Cell Counter (Invitrogen) was performed to monitor viable cell numbers. Extent of cell death was monitored by determination of LDH activity in culture medium using an LDH assay kit according to the manufacturer’s instructions.

Amounts of collagen in medium were measured with a Sircol assay kit according to the manufacturer’s protocol.

### Histological and immunohistochemical analyses

Lung tissue samples were fixed in 10% formalin neutral buffer solution for 24 h, and then embedded in paraffin before being cut into 4-μm-thick sections.

For histological examination, sections were stained first with Mayer’s haematoxylin and then with 1% eosin alcohol solution (H&E staining). Samples were mounted with malinol and scanned using a NanoZoomer-XR digital slide scanner (Hamamatsu Photonics, Shizuoka, Japan).

For staining of collagen (Masson’s trichrome staining), sections were treated sequentially with solution A (5% w/v potassium dichromate and 5% w/v TCA), Weigert’s iron haematoxylin, solution B (1.25% w/v phosphotungstic acid and 1.25% w/v phosphomolybdic acid), 0.75% w/v Orange G solution, solution C (0.12% w/v xylidine ponceau, 0.04% w/v acid fuchsin, and 0.02% w/v azophloxin), 2.5% w/v phosphotungstic acid, and finally with aniline blue solution. Samples were mounted with malinol and scanned using a NanoZoomer-XR digital slide scanner. Ashcroft score was determined to quantify the severity of pulmonary fibrosis based on a previously described method^24^.

For immunohistochemical analysis of α-SMA, sections were blocked with 2.5% goat serum for 10 min, incubated for 12 h with an antibody against α-SMA (1:100 dilution) in the presence of 2.5% bovine serum albumin, and then incubated with Alexa Fluor 594 goat anti-rabbit immunoglobulin G (1:500 dilution) and DAPI (5 μg/ml) for 2 h. Samples were mounted with VECTASHIELD and inspected with a fluorescence microscope (Olympus DP71). Image J software (National Institutes of Health, Bethesda, MD) was used to calculate the percentage of α-SMA-positive area.

For immunohistochemical analysis of 8-OHdG, sections were incubated first with proteinase K for 20 min at 37 °C, then with 0.3% hydrogen peroxide for 5 min, and finally with 2.5% goat serum for 10 min. Sections were then incubated with an antibody against 8-OHdG (1:200 dilution) for 12 h, followed by incubation with a peroxidase-labelled polymer conjugated to goat anti-mouse immunoglobulins for 1 h. Next, 3,3′-diaminobenzidine was applied to sections, which were then incubated with Mayer’s haematoxylin. Finally, samples were mounted with malinol and inspected with a microscope (Olympus DP71). Definiens Tissue Studio® software (CTC Life Science Corporation, Tokyo, Japan) was used to analyse the number of 8-OHdG–positive cells.

### Measurement of lung mechanics and FVC

Measurement of lung mechanics was performed with a computer-controlled small-animal ventilator (FlexiVent; SCIREQ, Montreal, Canada), as previously described^9^. Mice were anaesthetised with chloral hydrate (500 mg/kg), a tracheotomy was performed, and an 8-mm-long section of metallic tube (outer and inner diameters of 1.27 mm and 0.84 mm, respectively) was inserted into the trachea. Mice were mechanically ventilated at a rate of 150 breaths/min, using a tidal volume of 8.7 ml/kg and a positive end-expiratory pressure of 2–3 cmH_2_O.

Total respiratory system elastance and tissue elastance were measured by snap shot and forced oscillation techniques, respectively. All data were analysed using FlexiVent software (version 5.3; SCIREQ, Montreal, Canada).

Determination of FVC was performed with the same computer-controlled small-animal ventilator connected to a negative pressure reservoir (SCIREQ, Montreal, Canada), as previously described^9^. Mice were anaesthetised, then tracheotomised and ventilated as described above. Lungs were inflated to 30 cm H_2_O over 1 s and held at this pressure. After 0.2 s, the pinch valve (connected to the ventilator) was closed, and after 0.3 s, the shutter valve (connected to the negative pressure reservoir) was opened, exposing the lung to the negative pressure, which was held for 1.5 s to ensure complete expiration. FVC was determined using FlexiVent software (version 5.3).

### Hydroxyproline determination, and measurement of TBARS

Hydroxyproline content was determined as previously described^25^. Briefly, the lung was removed and homogenised in 0.5 ml of 5% TCA. After centrifugation, pellets were hydrolysed in 0.5 ml of 10 N HCl for 16 h at 110 °C. Each sample was incubated for 20 min at room temperature with 0.5 ml of 1.4% w/v chloramine T solution, and then incubated at 65 °C for 10 min with 0.5 ml of Ehrlich’s reagent (1 M DMBA, 70% v/v isopropanol and 30% v/v perchloric acid). Absorbance was measured at 550 nm to determine the amount of hydroxyproline.

Amounts of TBARS in the lung were measured with a TBARS assay kit according to the manufacturer’s protocol.

### Statistical analysis

All values are expressed as mean ± S.E.M. One-way ANOVA followed by Dunnett’s test or Welch’s *t*-test for unpaired results was used to evaluate differences between three or more groups or between two groups, respectively. SPSS22 software was used for all statistical analyses. Differences were considered to be significant for values of *P* *<* 0.05.

## Results

### Effect of idebenone on cell growth and death in LL29 and A549 cells

From a library of medicines already in clinical use, we screened for compounds eliciting cytotoxicity to lung fibroblasts but not lung epithelial cells. LL29 or A549 cells were exposed to each drug for 24 h and viable cell numbers were determined by the MTT method. Amongst drugs that showed lower IC_50_ values (concentration required for 50% decrease of viable cell number) for LL29 cells than for A549 cells, we selected idebenone based on the extent of difference of IC_50_ values between these two types of cells, clinical safety, and other pharmacological activity. Idebenone is a synthetic analogue of CoQ_10_ (antioxidant) and had been used clinically as a brain metabolic stimulant^26,27^.

**Fig. 1:**
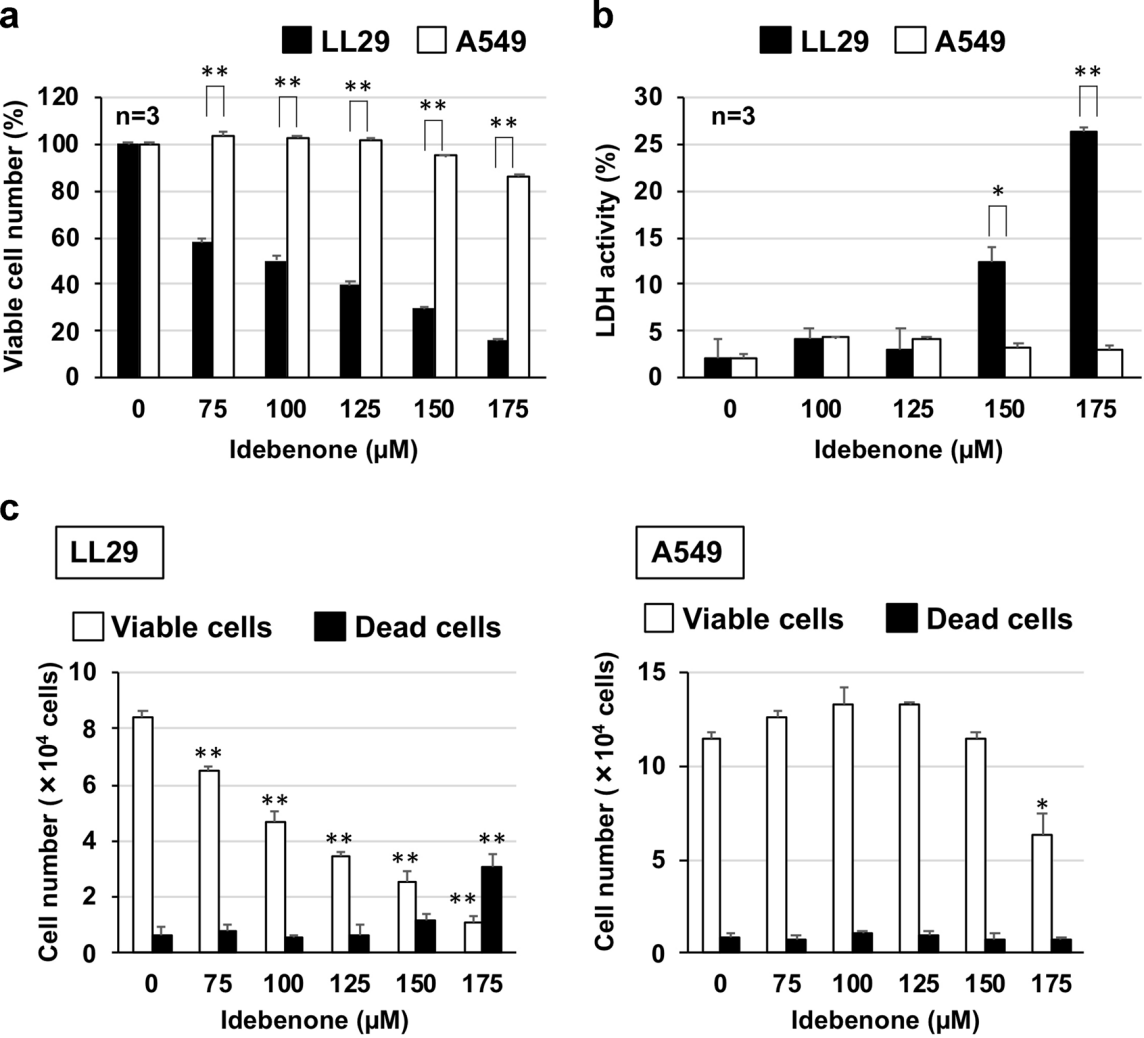
Cytotoxicity of idebenone in LL29 and A549 cells. LL29 or A549 cells were incubated with the indicated concentration of idebenone for 24 h (**a**, **b**). Viable cell number was determined by MTT method (**a**). Cell death was monitored by determination of LDH activity in culture medium (**b**). LL29 or A549 cells were pre-incubated for 24 h (from day 0 to day 1) and further incubated with the indicated concentration of idebenone for 24 h (from day 1 to day 2). Forty-eight hours after pre-culture, viable and dead cell numbers were monitored by trypan blue staining assay (**c**). Values represent mean ± S.E.M. **P < 0.01; *P < 0.05

As shown in Fig. 1a, treatment with idebenone decreased the number of viable LL29 cells at lower concentrations compared with A549 cells. To monitor cell death, LDH release from cells was monitored. The apparent release of LDH into the culture medium was observed at idebenone concentrations of 150 or 175 µM in LL29 cells. In contrast, LDH release was not observed in A549 cells at the same concentrations, suggesting that idebenone induces cell death preferentially in LL29 cells compared with A549 cells (Fig. 1b). Results shown in Fig. 1a, b also suggest that idebenone inhibits cell growth at concentrations 75–125 µM in LL29 cells. Direct cell counting by trypan blue staining revealed that idebenone at more than 75 µM suppressed the growth of LL29 cells, while concentrations greater than 175 µM induced cell death (Fig. 1c). Results also showed that idebenone induced cell growth inhibition at 175 µM, but did not induce cell death at 175 µM in A549 cells.

**Fig. 2:**
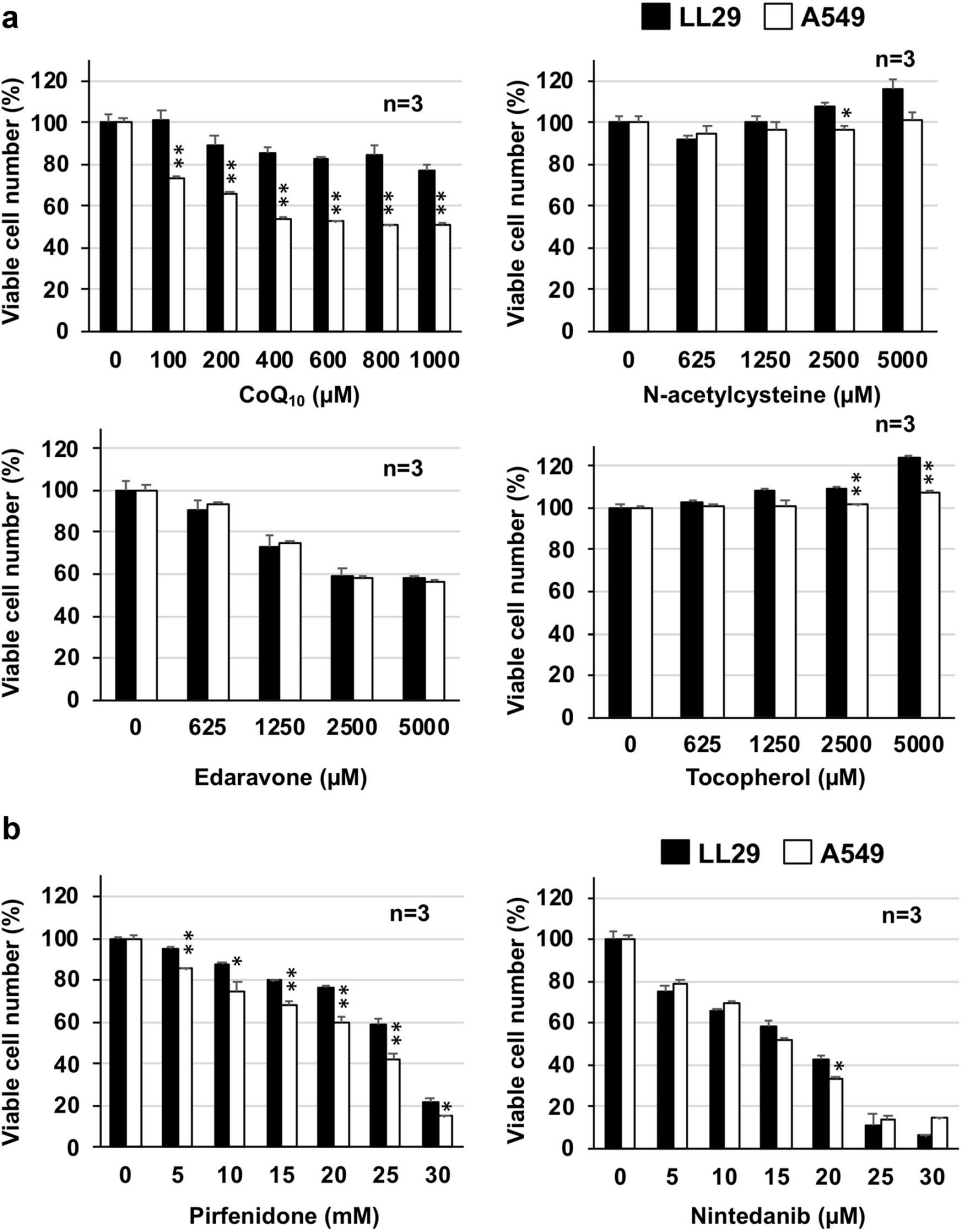
Cytotoxicity of other drugs in LL29 and A549 cells. LL29 or A549 cells were incubated with indicated concentrations of CoQ_10_, N-acetylcysteine, edaravone, α-tocopherol (**a**), pirfenidone or nintedanib (**b**) for 24 h. Viable cell number was determined by MTT method. Values represent mean ± S.E.M. **P < 0.01; *P < 0.05

To examine the specificity of idebenone for characteristics shown in Fig. 1, we examined effects of various drugs on viable cell numbers of both LL29 and A549 cells. As shown in Fig. 2, treatment with CoQ_10_ decreased the number of viable A549 cells at lower concentrations compared with LL29 cells, in contrast to the effect of idebenone. Other antioxidant molecules (NAC, edaravone and α-tocopherol) did not show preferential cytotoxicity for LL29 cells compared with A549 cells (Fig. 2a). Higher concentrations of NAC and α-tocopherol could not be tested because of their low solubility. These results suggest that the ROS-decreasing activity of idebenone is not involved in the preferential cytotoxicity for LL29 cells over A549 cells. Results shown in Fig. 2b also indicate that neither pirfenidone nor nintedanib (two recently approved drugs) show preferential cytotoxicity for LL29 cells compared with A549 cells.

### Effect of idebenone on bleomycin-induced pulmonary fibrosis

**Fig. 3:**
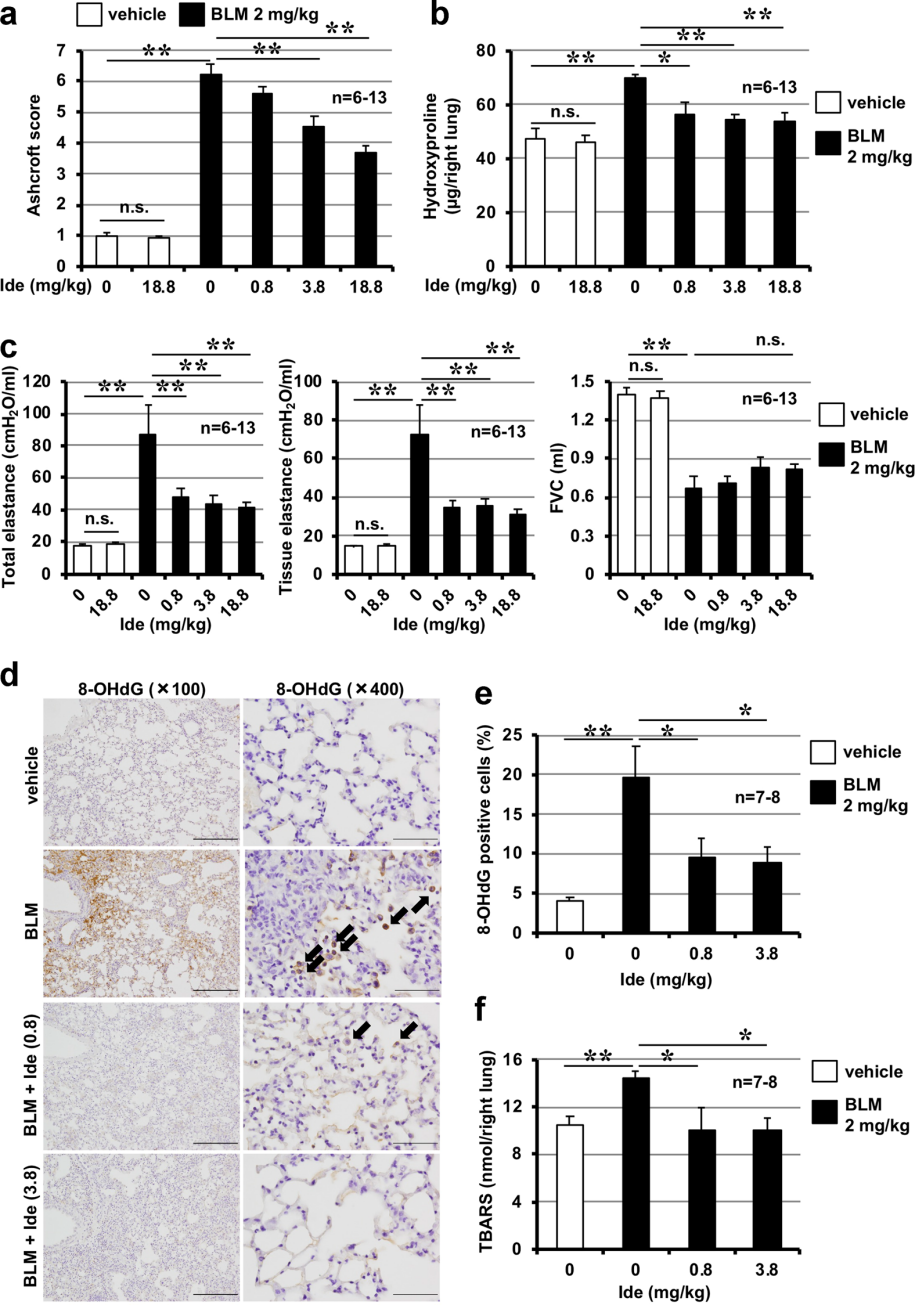
Effect of idebenone on bleomycin-induced pulmonary fibrosis, alteration of lung mechanics, respiratory dysfunction, and increases in oxidative stress. Mice were treated with bleomycin (BLM, 2 mg/kg) or vehicle once only on day 0. Mice were intratracheally administered indicated dose of idebenone (Ide) once daily for 8 days (from day 0 to day 7). Ashcroft score was determined based on the image of Masson’s trichrome staining (**a**). Pulmonary hydroxyproline level was determined on day 14 (**b**). Total respiratory system elastance, tissue elastance and FVC was measured on day 14 (**c**). Sections of pulmonary tissue were prepared on day 8 and subjected to immunohistochemical analysis with an antibody against 8-OHdG (scale bar = 200 μm (left image) or 50 μm (right image)] (**d**) and the number of 8-OHdG–positive cells was determined using Definiens Tissue Studio® software (**e**). Pulmonary TBARS level was determined on day 8 (**f**). Values represent mean ± S.E.M. **P < 0.01; *P < 0.05; n.s., not significant

Pulmonary fibrosis was induced in mice intratracheally administered bleomycin. Histopathological analysis of pulmonary tissue by H&E staining and Masson’s trichrome staining of collagen showed that bleomycin administration induced severe pulmonary damage (thickened and oedematous alveolar walls and interstitium) and collagen deposition; moreover, these lesions were suppressed by intratracheal administration of idebenone (Supplementary Fig. S1). Idebenone-dependent suppression of bleomycin-induced pulmonary fibrosis was also confirmed by determination of Ashcroft score (based on the image of Masson’s trichrome staining) (Fig. 3a) and pulmonary hydroxyproline content, two indicators of pulmonary fibrosis (Fig. 3b).

Changes in lung mechanics associated with pulmonary fibrosis are characterised by an increase in elastance^28^. Total respiratory system elastance (elastance of the total lung including bronchi, bronchioles and alveoli) and tissue elastance (elastance of the alveoli) increased following bleomycin treatment, and were partially restored by administration of idebenone (Fig. 3c). Using a computer-controlled ventilator and negative pressure reservoir, we found that FVC decreased in bleomycin-treated mice, while treatment with idebenone tended to suppress this decrease, although this was not statistically significant (Fig. 3c). These results demonstrate that intratracheal administration of idebenone protected against bleomycin-induced pulmonary fibrosis and alteration of lung mechanics. Moreover, these results show that intratracheal administration of idebenone (18.8 mg/kg) did not affect levels of pulmonary fibrosis, lung elastance and FVC in mice without bleomycin administration (Fig. 3a–c).

As described in the introduction, ROS play an important role in pulmonary fibrosis, and idebenone has ROS-decreasing activity^27^. Thus, we examined the effect of idebenone on bleomycin-induced increases in ROS levels by monitoring pulmonary levels of 8-OHdG and TBARS, two indicators of tissue ROS levels. As shown in Fig. 3d–f, treatment with bleomycin increased the pulmonary level of 8-OHdG and TBARS, and these increases were significantly suppressed by idebenone administration.

**Fig. 4:**
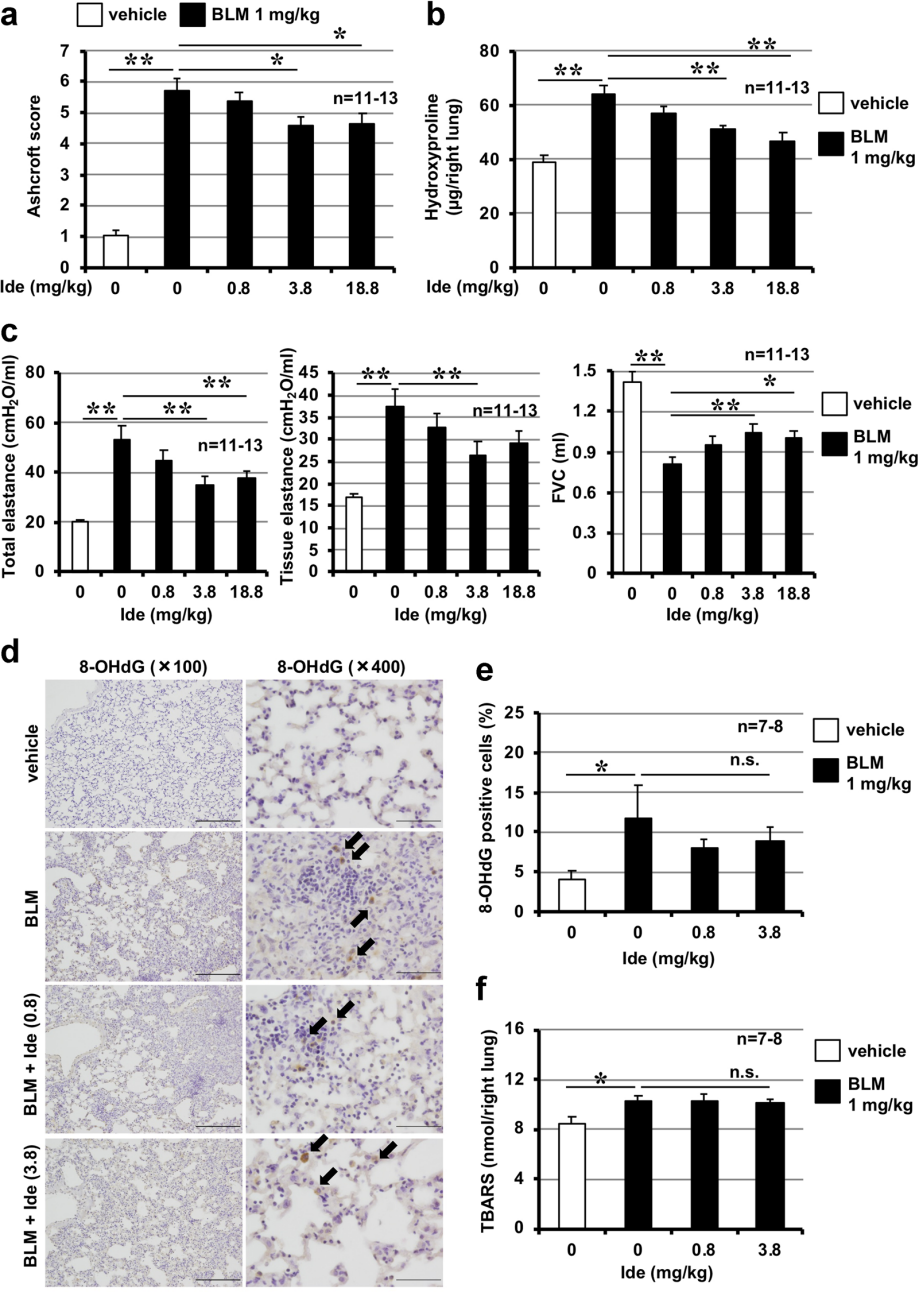
Effect of idebenone on pre-developed pulmonary fibrosis. Mice were treated with bleomycin (BLM, 1 mg/kg) or vehicle once only on day 0. Mice were intratracheally administered indicated dose of idebenone (Ide) once daily for 9 days (from day 10 to day 18) (**a**–**c**) or 3 days (from day 10 to day 12) (**d**–**f**). Ashcroft score was determined based on the image of Masson’s trichrome staining (**a**). Pulmonary hydroxyproline level was determined on day 20 (**b**). Total respiratory system elastance, tissue elastance and FVC were measured on day 20 (**c**). Sections of pulmonary tissue were prepared on day 13 and subjected to immunohistochemical analysis with an antibody against 8-OHdG [scale bar = 200 μm (left image) or 50 μm (right image)] (**d**) and the number of 8-OHdG–positive cells was determined using Definiens Tissue Studio® software (**e**). Pulmonary TBARS level was determined on day 13 (**f**). Values represent mean ± S.E.M. **P < 0.01; *P < 0.05; n.s., not significant

We next tested the efficacy of idebenone when the treatment protocol was started after the development of fibrosis. Drug treatment was commenced 10 days after bleomycin administration, and pulmonary fibrosis, lung mechanics and FVC were assessed on day 20. We first confirmed the presence of pulmonary fibrosis on day 10 (data not shown). As shown in Supplementary Fig. S2, Fig. 4a, b, administration of idebenone decreased the extent of pulmonary damage and fibrosis. We also found that idebenone administration significantly decreased lung elastance and increased FVC on day 20 (Fig. 4c). These data suggest that idebenone could be an effective agent for the treatment of pre-existing pulmonary fibrosis. However, administration of idebenone after the development of fibrosis did not affect pulmonary levels of 8-OHdG and TBARS (Fig. 4d–f), suggesting that the ROS-decreasing activity of this drug is not involved in its therapeutic effect for pulmonary fibrosis and lung functions.

### Comparison of idebenone and CoQ_10_ for bleomycin-induced pulmonary fibrosis and increases in myofibroblasts

**Fig. 5:**
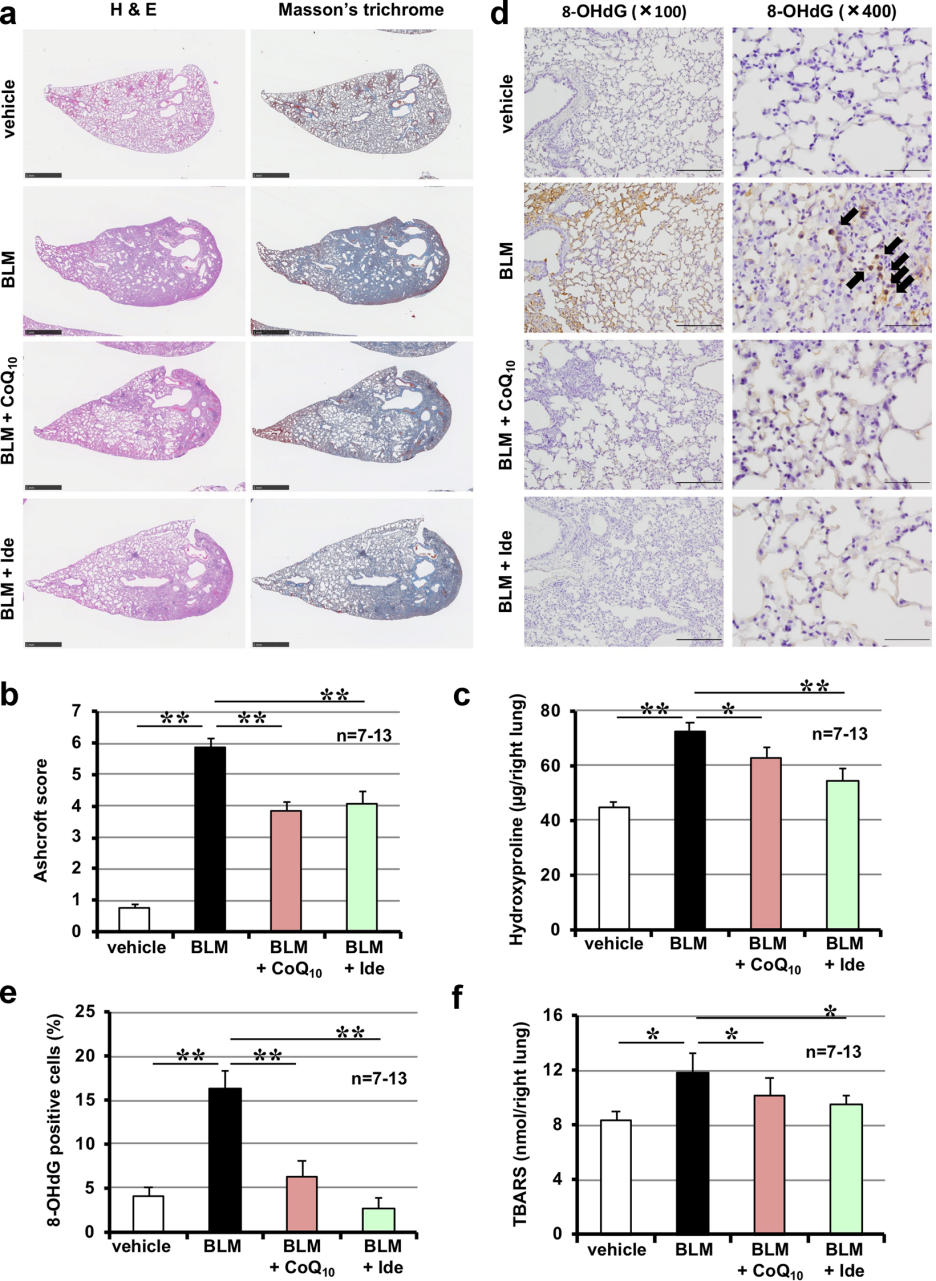
Comparison of idebenone and CoQ_10_ for bleomycin-induced pulmonary fibrosis. Mice were treated with bleomycin (BLM, 2 mg/kg) or vehicle once only on day 0. Mice were intratracheally administered idebenone (Ide, 3.8 mg/kg) or CoQ_10_ (9.6 mg/kg) once daily for 8 days (from day 0 to day 7). Sections of pulmonary tissue were prepared on day 14 and subjected to histopathological examination (H&E staining and Masson’s trichrome staining; scale bar = 1.0 mm) (**a**). Ashcroft score was determined based on the image of Masson’s trichrome staining (**b**). Pulmonary hydroxyproline level was determined on day 14 (**c**). Sections of pulmonary tissue were prepared on day 8 and subjected to immunohistochemical analysis with an antibody against 8-OHdG [scale bar = 200 μm (left image) or 50 μm (right image)] (**d**) and the number of 8-OHdG–positive cells was determined using Definiens Tissue Studio® software (**e**). Pulmonary TBARS level was determined on day 8 (**f**). Values represent mean ± S.E.M. **P < 0.01; *P < 0.05

To understand the mechanism governing protective and therapeutic effects of idebenone for bleomycin-induced pulmonary fibrosis, we compared its activity with CoQ_10_. We used a dosage of CoQ_10_ (9.6 mg/kg) that is equivalent to idebenone (3.8 mg/kg) with regard to number of molecule. As shown in Fig. 5a–c, bleomycin-induced pulmonary damage and fibrosis were suppressed by administration of either idebenone or CoQ_10_. Furthermore, administration of idebenone or CoQ_10_ suppressed bleomycin-induced increases in pulmonary levels of 8-OHdG and TBARS (Fig. 5d–f).

**Fig. 6:**
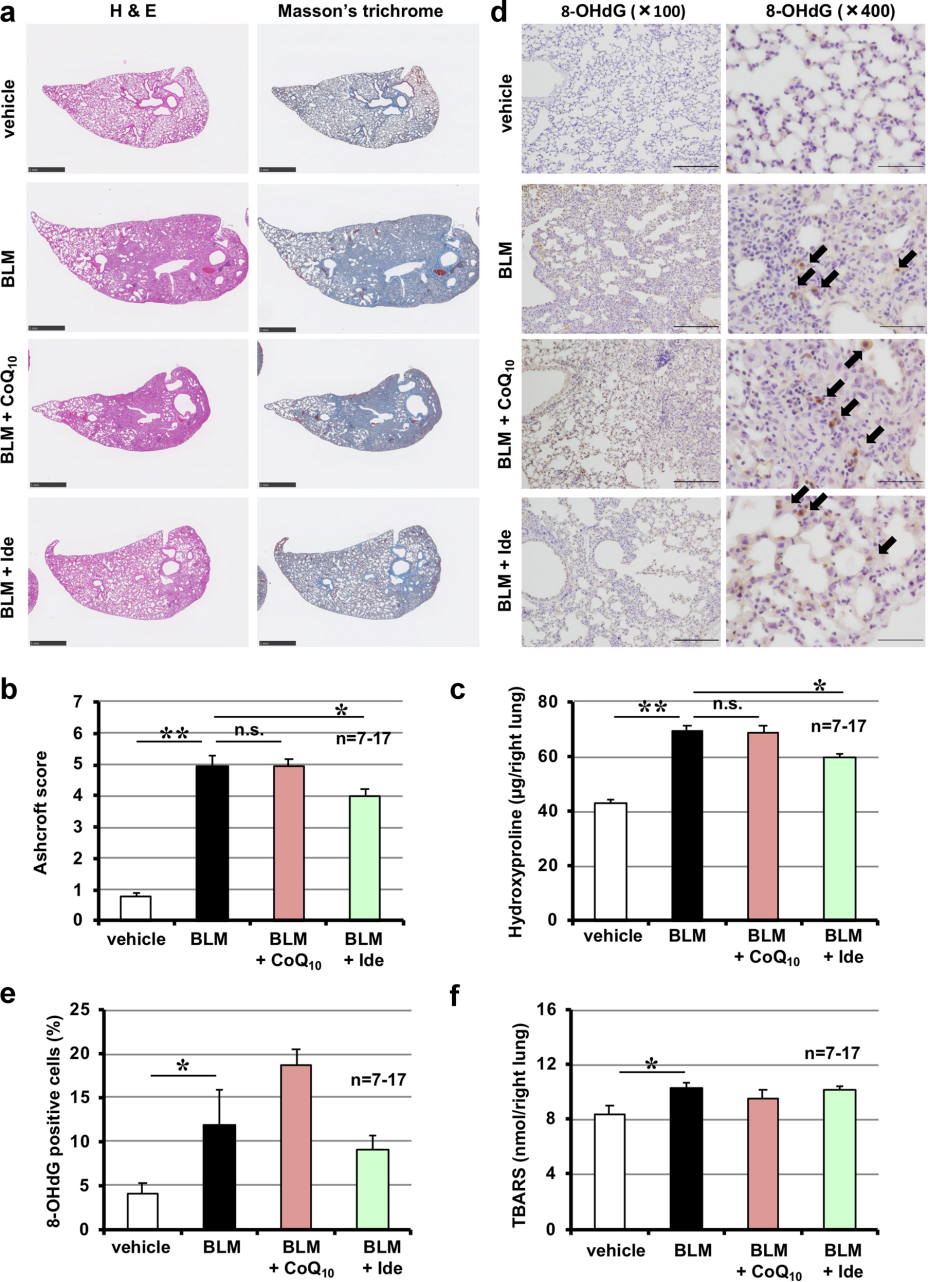
Comparison of idebenone and CoQ_10_ for pre-developed pulmonary fibrosis. Mice were treated with bleomycin (BLM, 1 mg/kg) or vehicle once only on day 0. Mice were intratracheally administered idebenone (Ide, 3.8 mg/kg) or CoQ_10_ (9.6 mg/kg) once daily for 9 days (from day 10 to day 18) (**a**–**c**) or 3 days (from day 10 to day 12) (**d**–**f**). Sections of pulmonary tissue were prepared on day 20 and subjected to histopathological examination (H&E staining and Masson’s trichrome staining; scale bar = 1.0 mm) (**a**). Ashcroft score was determined based on the image of Masson’s trichrome staining (**b**). Pulmonary hydroxyproline level was determined on day 20 (**c**). Sections of pulmonary tissue were prepared on day 13 and subjected to immunohistochemical analysis with an antibody against 8-OHdG [scale bar = 200 μm (left image) or 50 μm (right image)] (**d**) and the number of 8-OHdG–positive cells was determined using Definiens Tissue Studio® software (**e**). Pulmonary TBARS level was determined on day 13 (**f**). Values represent mean ± S.E.M. **P < 0.01; *P < 0.05

We also compared pharmacological activity between idebenone and CoQ_10_ for pre-developed fibrosis. As shown in Fig. 6a–c, idebenone but not CoQ_10_, affected bleomycin-induced pulmonary damage and fibrosis. In contrast, neither idebenone nor CoQ_10_ affected pulmonary levels of 8-OHdG and TBARS (Fig. 6d–f). Results shown in Figs. 5 and 6 suggested that CoQ_10_ could not affect pulmonary fibrosis when administered after the development of fibrosis, and this difference between idebenone and CoQ_10_ could not be explained by their ROS-decreasing activities.

**Fig. 7:**
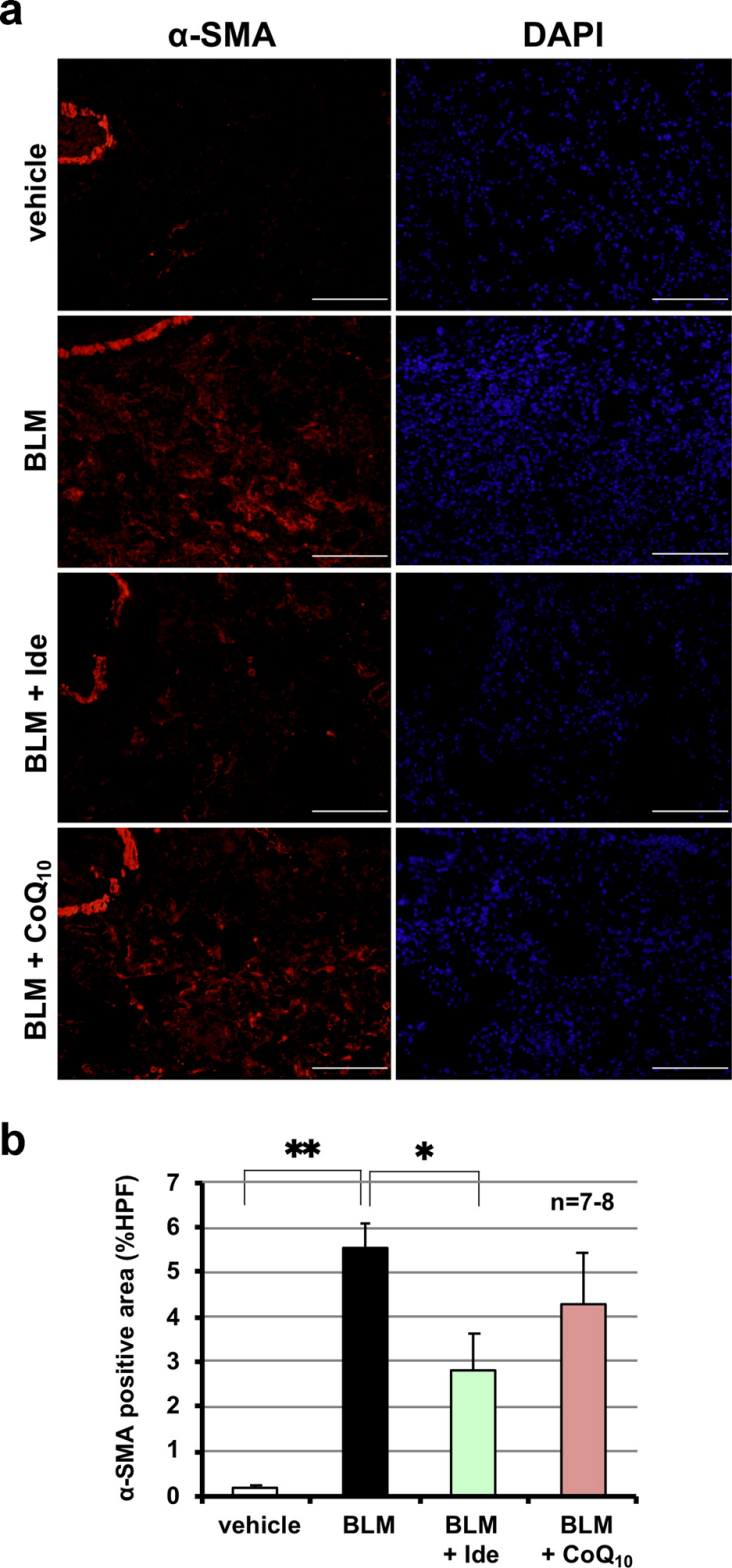
Comparison of idebenone and CoQ_10_ for bleomycin-induced increases in myofibroblasts. Mice were treated with bleomycin (BLM, 1 mg/kg) or vehicle once only on day 0. Mice were intratracheally administered idebenone (Ide, 3.8 mg/kg) or CoQ_10_ (9.6 mg/kg) once daily for 3 days (from day 10 to day 12). Sections of pulmonary tissue were prepared on day 13 and subjected to immunohistochemical analysis with an antibody against α−SMA (scale bar = 100 μm) (**a**). Percentage of area stained with the antibody was determined using ImageJ software (**b**). Values represent mean ± S.E.M. **P < 0.01; *P < 0.05

As described in the introduction, myofibroblasts play an important role in pulmonary fibrosis in IPF patients and bleomycin-induced pulmonary fibrosis in animals^11,29^. Thus, we monitored levels of myofibroblasts by immunostaining for α-SMA, a marker of myofibroblasts^11^. As shown in Fig. 7, administration of bleomycin increased the number of α-SMA–positive cells in the lung and idebenone, but not CoQ_10_, suppressed this increase, suggesting that idebenone, but not CoQ_10_, suppressed transdifferentiation of fibroblasts into myofibroblasts.

**Fig. 8:**
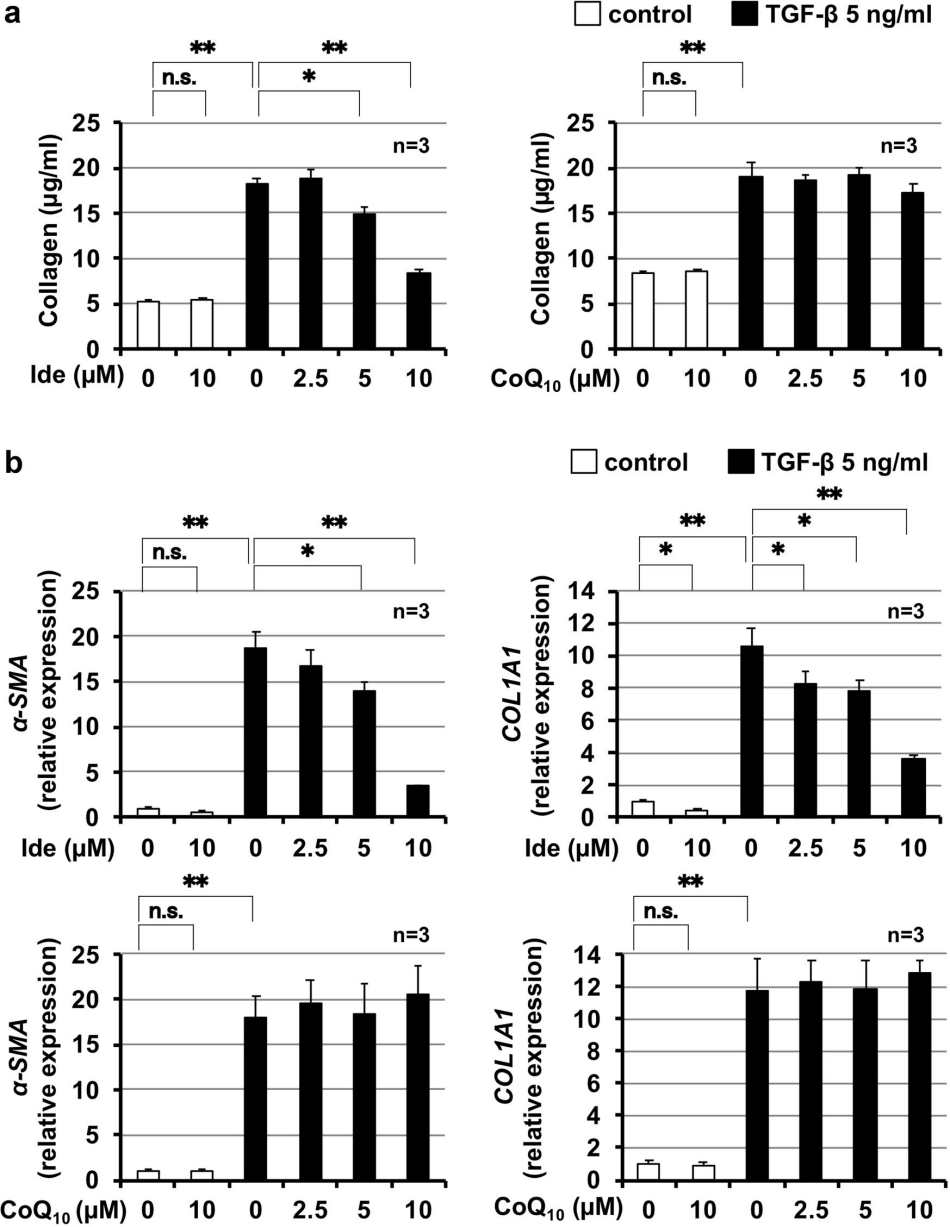
Comparison of idebenone and CoQ_10_ for TGF-β1-induced collagen production. LL29 cells were incubated with TGF-β1 (5 ng/ml) for 48 h (**a**) or 24 h (**b**) in the presence of the indicated concentration of idebenone (Ide) or CoQ_10_ (**b**). Level of collagen in the culture medium was determined by Sircol assay (**a**). Total RNA was extracted and subjected to real-time RT-PCR using a specific primer set for each gene. Values were normalised to *Gapdh* gene expression, and expressed relative to the control sample (**b**). Values represent mean ± S.E.M. **P < 0.01; *P < 0.05; n.s., not significant

Thus, we compared the effect of idebenone and CoQ_10_ on TGF-β1–induced activation of lung fibroblasts. LL29 cells were treated with idebenone or CoQ_10_ in the presence of TGF-β1 and then fibroblast activation was monitored by level of collagen within the culture media. As shown in Fig. 8a, treatment with TGF-β1 increased collagen levels, and idebenone, but not CoQ_10_, suppressed this increase. Activation of lung fibroblasts was also tested by monitoring α-SMA mRNA expression and collagen. As shown in Fig. 8b, treatment of LL29 cells with TGF-β1-induced expression of α*-SMA* and *Col1a1* mRNA, while simultaneous treatment of cells with idebenone, but not CoQ_10_, suppressed this induction. These results suggest that idebenone suppressed TGF-β1–induced activation of lung fibroblasts in vitro.

## Discussion

In this study, we identified idebenone from medicines already in clinical use as a compound that can preferentially inhibit the growth of lung fibroblasts compared with lung alveolar epithelial cells. Administration of idebenone suppressed bleomycin-induced pulmonary fibrosis, alteration of lung mechanics, and increases in pulmonary ROS levels. Further, idebenone had an inhibitory activity on the function of lung fibroblasts both in vivo and in vitro. These results suggest that both suppression of ROS levels and inhibition of lung fibroblast function by idebenone treatment contribute to its inhibitory effect on pulmonary fibrosis.

To the best of our knowledge, this is the first study to report the therapeutic effect of idebenone against bleomycin-induced pulmonary fibrosis, a representative animal model of IPF. To develop a new IPF drug, it is important to examine not only its preventive effect but also its therapeutic effects in an animal model of IPF. Further, two recently developed IPF drugs, pirfenidone and nintedanib, have been reported to have therapeutic effects on bleomycin-induced pulmonary fibrosis^15,30^. Moreover, as the diagnosis of IPF in human patients is confirmed by a decrease in FVC^2^, it is important to examine the effect of a candidate drug on BLM-dependent respiratory failure, especially a decrease in FVC. Thus, we examined the therapeutic effect of idebenone and the effect of idebenone on bleomycin-induced decreases in FVC in this study (Fig. 4). As mentioned in the Results section, idebenone clearly showed both therapeutic and improving effects against BLM-dependent decreases in FVC. We therefore assume that idebenone may have therapeutic benefit for IPF patients in addition to pirfenidone and nintedanib.

While both pirfenidone and nintedanib significantly improved the reduction of FVC in clinical trials of IPF patients^2,5,6^, and were already approved, they were also reported to have severe adverse effects, such as dyspepsia and diarrhoea in clinical setting^5,6^. Thus, we employed a “drug repositioning strategy” in this study to discover safer drugs for IPF treatment, with the advantage of this strategy being that the safety of approved drugs is already well understood. Furthermore, as shown in Fig. 1, idebenone preferentially inhibited the growth of lung fibroblasts compared with lung alveolar epithelial cells. In contrast, neither pirfenidone nor nintedanib showed preferential cytotoxicity for lung fibroblasts over lung alveolar epithelial cells (Fig. 2). Considering that idebenone preferentially suppresses the action of fibroblasts, which are the cause of the onset of IPF, it is highly expected that idebenone has fewer side effects than these two approved IPF drugs.

Regarding the anti-fibrotic mechanisms of idebenone, we revealed that both of suppression of ROS levels and inhibition of lung fibroblast activity by idebenone contribute to its inhibitory effect on pulmonary fibrosis. As we did not examine the detailed mechanism by which idebenone suppressed ROS levels or preferentially inhibited the growth of lung fibroblasts in this study, these mechanisms need to be clarified to identify the primary target of idebenone. Idebenone, a synthetic analogue of CoQ_10_, interacts with the mitochondrial electron transport chain^26,27^. Thereby, idebenone has been reported to increase a major energy (ATP) production in mitochondria and suppress lipid peroxidative reactions^31^. Moreover, idebenone directly suppressed glycerophosphate-dependent ROS production in vitro^32^. We suggest that idebenone suppresses bleomycin-dependent ROS increases by these mechanisms. In contrast, the mechanism by which idenenone preferentially inhibited the growth of lung fibroblasts is unknown. A recent study suggested that syndecan-2 attenuates radiation-induced pulmonary fibrosis and inhibits fibroblast activation by regulating the PI3K/Akt/ROCK pathway via CD148^33^. Tsukui et al. have identified osteopontin as the most highly expressed gene in fibroblasts, and suggested that osteopontin may serve as a useful marker of profibrotic fibroblasts^34^. Moreover, Goodwin et al. showed that targeting the HIF-1α/PDK1 axis suppresses bleomycin-induced pulmonary fibrosis by suppressing myofibroblast differentiation and fibroblast progression^35^. Further, miR-101 reportedly attenuates bleomycin-induced pulmonary fibrosis by inhibiting fibroblast proliferation and activation^36^. Based on these reports, we assume that these mechanisms are involved in preferential inhibition of lung fibroblasts by idebenone.

Considering the clinical application of idebenone, it is important to examine the route of administration of idebenone in IPF animal models. Both pirfenidone and nintedanib are administered orally in tablets for treating IPF patients. Idebenone was approved for use as an orally administered drug as a brain metabolic stimulant^26,27^. In contrast, we examined the effect of intratracheally administered idebenone on bleomycin-induced pulmonary fibrosis in this study because idebenone is likely to directly suppress the action of pulmonary fibroblasts. In addition, clinical trials have been conducted to investigate the administration of several drugs (such as NAC and interferon) directly to the lungs^37,38^. Based on this background and because examining the route of administration is highly worthwhile, we hope to examine the route of idebenone administration in IPF animal models in future experiments.

Based on all findings in this study, we propose that idebenone may be therapeutically beneficial for IPF patients, as safety for use in humans has already been clinically confirmed.

## Supplementary information

SUpple
