# Supplementary material for: Idebenone has preventative and therapeutic effects on pulmonary fibrosis via preferential suppression of fibroblast activity
Source: Cell Death Discov. 2019 Nov 18;5:146. doi: 10.1038/s41420-019-0226-y (PMC6861265; doi:10.1038/s41420-019-0226-y)
Supplement: Supplementary file 1 — SUpple [file 41420_2019_226_MOESM1_ESM.pdf]

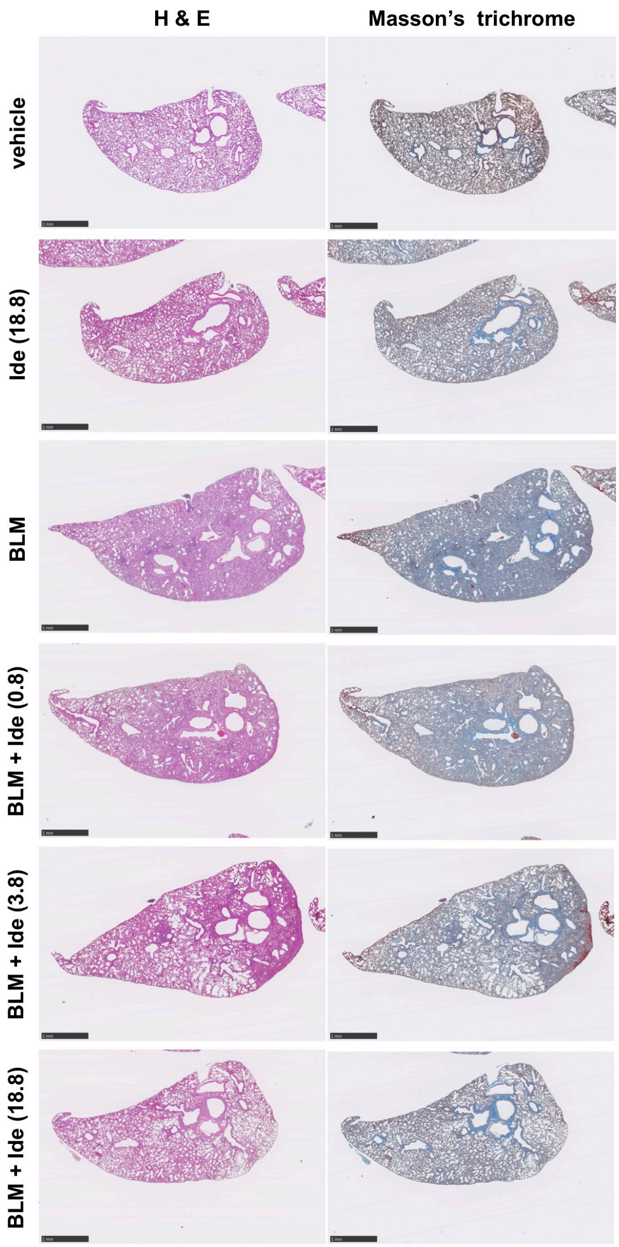

Supplementary Fig. S1. Effect of idebenone on bleomycin-induced pulmonary fibrosis.

Mice were treated with bleomycin (BLM, 2 mg/kg) or vehicle once only on day 0. Mice were intratracheally administered indicated dose of idebenone (Ide) once daily for 8 days (from day 0 to day 7). Sections of pulmonary tissue were prepared on day 14 and subjected to histopathological examination (H&E staining and Masson’s trichrome staining; scale bar = 1.0 mm).

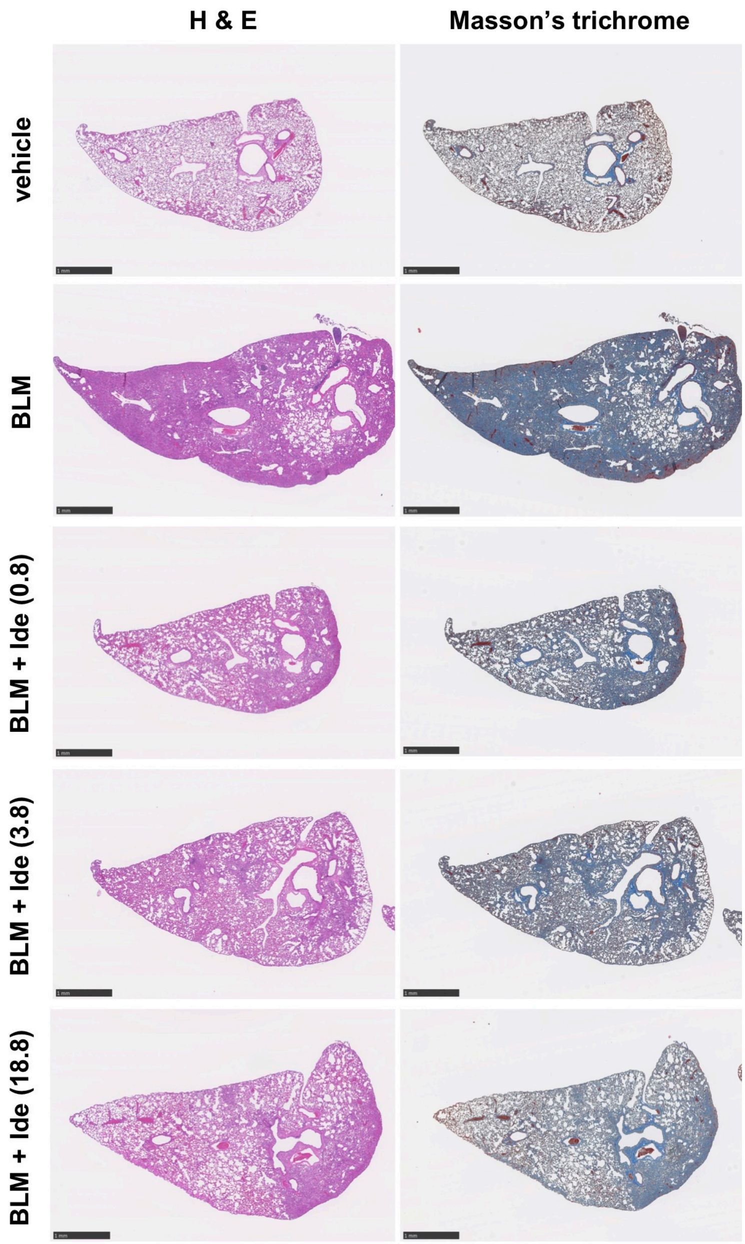

Supplementary Fig. S2. Effect of idebenone on pre-developed pulmonary fibrosis. Mice were treated with bleomycin (BLM, 1 mg/kg) or vehicle once only on day 0. Mice were intratracheally administered indicated dose of idebenone (Ide) once daily for 9 days (from day 10 to day 18). Sections of pulmonary tissue were prepared on day 20 and subjected to histopathological examination (H&E staining and Masson's trichrome staining; scale bar = 1.0 mm). A
